# The addition of bevacizumab to standard chemotherapy in breast cancer: which patient benefits the most?

**DOI:** 10.1186/2193-1801-2-202

**Published:** 2013-05-01

**Authors:** Vibeke Kruse, Hannelore Denys, Rudy Van Den Broecke, Simon Van Belle, Veronique Cocquyt

**Affiliations:** Department of Medical Oncology, University Hospital Ghent, De Pintelaan 185, 9000 Ghent, Belgium; Heymans Institute of Pharmacology, University of Ghent, Ghent, Belgium; Department of Gynaecology, University Hospital Ghent, Ghent, Belgium

**Keywords:** Breast cancer, Bevacizumab, Age, Metastases, Survival, Safety

## Abstract

Bevacizumab, a humanized monoclonal antibody directed against vascular endothelial growth factor, is an effective and well-tolerated treatment option for patients with breast cancer. Bevacizumab has demonstrated a gain in progression-free survival and a trend towards an overall survival benefit in various subgroups of breast cancer. Given the lack of a predictive biomarker, we performed a literature search with regard to efficacy and tolerability of bevacizumab in different subgroups of breast cancer patients and in different settings. In the metastatic setting, the efficacy of bevacizumab has been most extensively studied and demonstrated in patients with triple-negative breast cancer, the most difficult-to-treat population among patients with advanced disease and also the group with the biggest need for new treatment options. Overall, bevacizumab is well tolerated with very few serious adverse events. Bevacizumab is also an active and feasible treatment option for patients above 70 years of age.

## Introduction

Breast cancer (BC) is a heterogeneous disease with varied molecular features, morphological subtypes, and differential response to therapy. Standard treatment for metastatic breast cancer (MBC) is hormonal therapy or chemotherapy. Approximately 20–25*%* of early-stage BCs over-express human epidermal growth factor receptor 2 (HER2) and can be treated with HER2-targeted agents (e.g., trastuzumab and lapatinib) (Pegram et al. 1998). The most active chemotherapeutic agents are taxanes and anthracyclines, with activity also seen with vinorelbine, capecitabine, gemcitabine, and eribulin (Lohman & Chia 2012). The combination of anti-angiogenic agents (e.g., bevacizumab) with chemotherapy has demonstrated longer progression-free survival (PFS) with acceptable toxicity.

Angiogenesis is critical for tumor growth and the development of metastatic disease. Vascular endothelial growth factor (VEGF) is an angiogenic mediator that stimulates endothelial cell proliferation and migration; inhibits endothelial cell apoptosis; induces extracellular matrix remodeling; increases vascular permeability; and inhibits antigen-presenting dendritic cells. Tumors with enhanced angiogenesis, supporting rapid growth and early metastases (e.g., triple-negative breast cancer [TNBC]), have high levels of VEGF (Greenberg & Rugo 2010).

Bevacizumab (Genentech Inc, CA, U.S.) is a humanized monoclonal antibody against all VEGF-A isoforms. Bevacizumab prevents binding of VEGF to receptors on vascular endothelial cells, leading to inhibition of angiogenesis and tumor growth. A Phase I/II clinical trial investigated 3, 10, or 20 mg/kg of bevacizumab monotherapy in patients with previously treated MBC. The objective response rate (ORR) was 9.3*%*, and 17*%* of patients had a response or were stable at 22 weeks; 10 mg/kg was suggested for further trials (Cobleigh et al. 2003). Bevacizumab may also potentiate the effect of chemotherapy regimens.

## Search strategy

English language reports of bevacizumab clinical trials in BC were identified by searching PubMed and the American Society of Clinical Oncology and European Society of Medical Oncology databases using the following terms: ‘bevacizumab’ and ‘breast cancer’ or ‘tolerance’ or ‘chemotherapy’ or ‘review’ or ‘safety’ or ‘angiogenesis’. Additional relevant studies were found via bibliographic review of reports identified during the systematic search. Studies in patients with locally recurrent (LR) or MBC who received bevacizumab in combination with standard chemotherapy with an appropriate trial design, appropriate statistical methodology, and a sufficient number of patients were included, as well as relevant pharmacodynamic and pharmacokinetic data.

## Results

### Data selection

We identified 14 papers presenting data from large, randomized, Phase III trials and two papers presenting data from Phase I/II trials on bevacizumab in BC. We also refer to three abstracts presented at The Annual San Antonio Breast Cancer Symposium and The Annual Congress of the European Society for Medical Oncology, including data from two meta-analyses. Furthermore, we selected one Cochrane Review, one pooled analysis of bevacizumab efficacy, and one National Cancer Institute report on bevacizumab in BC. We also identified 13 reviews on safety, pathological issues, angiogenesis, and biomarker considerations with respect to bevacizumab.

### First-line HER2-negative LR/MBC

In the first-line setting, E2100 (*n* = 685) demonstrated significant PFS and ORR improvements with paclitaxel plus bevacizumab compared with paclitaxel alone among patients with HER2-negative LR/MBC (Miller et al. 2007). Median PFS was approximately 11.4 months with the combination versus 5.8 months with paclitaxel alone (Gray et al. 2009). ORR was also significantly higher with paclitaxel-bevacizumab than with paclitaxel alone (48.0*%* vs. 23.4*%*, *P* < 0.0001) (Gray et al. 2009). These results were validated by independent review (Gray et al. 2009). The AVADO trial (*n* = 736) evaluated the efficacy and safety of bevacizumab (7.5 and 15 mg/kg) plus docetaxel as first-line therapy for HER2-negative, LR/MBC (Miles et al. 2010a). Bevacizumab (15 mg/kg) plus docetaxel showed a 1.9-month (*P =* 0.06) improvement in median PFS compared with placebo plus docetaxel. Response rates in patients with measurable disease at baseline were also increased with bevacizumab (46*%* with placebo vs. 55*%* with bevacizumab 7.5 mg/kg [*P* = 0.07] and 64*%* with bevacizumab 15 mg/kg [*P* < 0.001]). In the RIBBON-1 trial (*n* = 1,237), the combination of bevacizumab with capecitabine, taxane-based, or anthracycline-based chemotherapy was evaluated as first-line treatment for HER2-negative MBC (Robert et al. 2011). Median PFS was longer for each bevacizumab-chemotherapy combination compared with placebo-chemotherapy, and was most pronounced in the capecitabine cohort where PFS increased from 5.7 months to 8.6 months (*P* = 0.0002). ORR in patients with measurable disease was significantly higher in the bevacizumab-containing arm compared with the placebo arm for each chemotherapy cohort. There was no significant difference in overall survival (OS) between the placebo- and bevacizumab-containing arms (Robert et al. 2011). The ATHENA trial (*n* = 2,251) evaluated the efficacy of bevacizumab in combination with taxane-based chemotherapy in routine oncology practice (Smith et al. 2011). Median treatment duration was 6.2 months (range 0.0–29.7) for bevacizumab and 4.2 months (range 0.0–29.5) for chemotherapy. The median time to progression (TTP) was 9.5 months (95*%* confidence interval [CI] 9.1 - 9.9). ORR (best response) was 52*%* in the intent-to-treat (ITT) population. A further 33*%* of patients achieved stable disease (Smith et al. 2011). Mature data from ATHENA demonstrated a median OS of 25.2 months in patients receiving bevacizumab plus standard first-line chemotherapy (Pritchard et al. 2010). Patients continuing single-agent bevacizumab until disease progression after stopping chemotherapy showed a median TTP of 11.6 months with median OS of 30.0 months. There was no relationship detected between development of hypertension and OS (Pritchard et al. 2010).

### Second-line HER2-negative LR/MBC

Several trials have investigated bevacizumab for previously treated MBC. RIBBON-2 (*n* = 684) evaluated the efficacy and safety of bevacizumab plus commonly used chemotherapies for the second-line treatment of MBC (Brufsky et al. 2011). Bevacizumab plus chemotherapy increased median PFS from 5.1 to 7.2 months (hazard ratio [HR] 0.78, 95*%* CI 0.64 - 0.93, *P* = 0.0072) and reduced the risk of death by 22*%* (HR 0.78, 95*%* CI 0.64 - 0.93, log rank *P* = 0.0072). The ORR improvement between the placebo- and bevacizumab-containing arms (39.5*%* and 29.6*%*, respectively; not significant) was consistent with previous trials. Results of the earlier AVF2119g trial are less encouraging (Miller et al. 2005). The AVF2119g trial evaluated the combination of bevacizumab with capecitabine, but failed to meet its primary endpoint of prolonged PFS (median 4.9 vs. 4.2 months for bevacizumab-capecitabine compared with capecitabine alone, respectively; HR 0.98). However, patients included in the AVF2119g study had a poor prognosis (Miller et al. 2005).

### Subgroup analyses

Phase III trials have established consistent improvements in PFS by combining bevacizumab with chemotherapy as first-line treatment for MBC. Improved PFS was also seen in the second-line setting. However, to date all trials have failed to demonstrate a clear survival benefit. We reviewed the subgroup analyses of the trials to detect which patients would most benefit from the addition of bevacizumab treatment.

#### TNBC (HER2-negative, estrogen-/progesterone-receptor negative)

The prognosis for patients with metastatic TNBC is typically poor and there is no established standard therapy. Taxane-based regimens are among the most active and represent a reasonable approach for TNBC patients with a high risk of relapse.

A meta-analysis of TNBC patients in the randomized E2100, AVADO, and RIBBON-1 trials (*n* = 363 treated with bevacizumab plus chemotherapy, *n* = 258 treated with chemotherapy alone) showed a significant improvement in PFS for patients treated with chemotherapy plus bevacizumab (O’Shaughnessy et al. 2010). Median PFS was 8.1 months with bevacizumab-containing therapy versus 5.4 months for chemotherapy alone (HR 0.680, *P* = 0.0002). The PFS benefit remained when the patient subgroups (disease-free interval ≤24 months vs. >24 months; number of metastatic sites <3 vs. ≥3; the presence or not of visceral metastases) were assessed. ORR was significantly higher with bevacizumab-containing therapy than with chemotherapy alone (42*%* vs. 23*%*, *P* < 0.0001) (O’Shaughnessy et al. 2010).

An exploratory subgroup analysis of TNBC patients treated during routine oncology practice in the ATHENA study confirmed that the combination of paclitaxel-bevacizumab was active. In this subgroup, the median TTP was 7.2 months (95*%* CI 6.6 - 7.8) and the ORR was 49*%*, with a complete response (CR) rate of 10*%*. The 1-year OS rate was 60*%*. Median OS was 18.3 months (TNBC subgroup; 95*%* CI 16.4–19.7), 27.3 months (non-TNBC subgroup), and 25.2 months (overall population) (Thomssen et al. 2012). Updated survival results demonstrated a median OS of 18.3 months in patients with TNBC.

A subgroup analysis of the RIBBON-2 trial of TNBC patients who had progressed on first-line non-bevacizumab-containing chemotherapy also revealed a significant benefit of adding bevacizumab to standard second-line chemotherapy (Brufsky et al. 2012). Median PFS was 6.0 months with bevacizumab-chemotherapy versus 2.7 months with chemotherapy alone (HR 0.494, 95*%* CI 0.33 - 0.74, *P* = 0.0006). ORR was 41*%* vs. 18*%*, respectively, (*P* = 0.0078). Despite the small sample size and immature data, there was a trend towards improved OS: 17.9 months versus 12.6 months, respectively (HR 0.624, 95*%* CI 0.39 - 1.007, *P* = 0.0534).

The safety profile of bevacizumab in the TNBC subgroup was consistent with previous reports.

#### Elderly patients with HER2-negative MBC

The AVADO trial included 127 elderly (≥65 years) patients with HER2-negative MBC (Pivot et al. 2011). PFS was increased with bevacizumab therapy in the elderly subpopulation, the effect being greater with the 15 mg/kg dose but not statistically significant (PFS 10.1 months vs. 7.7 months with 7.5 mg/kg, HR 0.68, 95*%* CI 0.428 - 1.076). PFS data were generally consistent with the overall study population. Elderly patients with measurable disease at baseline had a higher ORR with bevacizumab plus docetaxel compared with placebo plus docetaxel (50*%*, 95*%* CI 35.2 - 64.8 vs. 44.7*%*, 95*%* CI 28.6 - 61.5). In the overall ITT population, response rates were higher than in the elderly ITT subpopulation, with the higher dose bevacizumab (15 mg/kg) arm being statistically superior to the placebo group (64.1*%* vs. 46.6*%*, *P* < 0.001). For the 1-year survival rates, there was a statistically significant treatment effect for the overall bevacizumab ITT population versus the ITT placebo population (84*%* vs. 76*%*, respectively, *P* < 0.02). The safety profile of bevacizumab was consistent in the overall and elderly populations (Pivot et al. 2011). Given the risk of arterial thromboembolic events with bevacizumab, it has been recommended that patients aged ≥65 years receive prophylactic low-dose aspirin (Hamilton & Blackwell 2011).

The elderly subpopulation of the ATHENA study comprised 175 patients aged ≥70 years (Biganzoli et al. 2012). Almost half of the elderly patients (46*%*) received paclitaxel plus bevacizumab. Median TTP was 10.4 months among elderly patients compared with 9.5 months (95*%* CI 9.1 - 9.9) in the overall ITT population. ORR was 42*%* for the elderly patients and 52*%* in the overall ITT population. The combination was well tolerated by elderly patients. Only hypertension and proteinuria were more common in older than in younger patients (Grade ≥3 hypertension: 6.9*%* vs. 4.2*%*, respectively; Grade ≥3 proteinuria: 4.0*%* vs. 1.5*%*, respectively) (Biganzoli et al. 2012). Updated survival results showed a median OS of 20.4 months for elderly patients treated with combination therapy. Adverse events (AEs) of bevacizumab among patients aged ≥70 years versus patients aged <70 years are presented (Table 1) (Biganzoli et al. 2012).

**Table 1:** **Grade 3 to 5 adverse events of special interest in patients aged ≥70 years versus patients aged <70 years in the ATHENA trial (Biganzoli et al.**^2012^**) [Permission required for reproduction]**

	Age <70 years (***n*** = 2,076), ***%***		Age ≥70 years (***n*** = 175), ***%***	
	On treatment	Entire study period	On treatment	Entire study period
Hypertension	3.2	4.2	5.1	6.9
Grade 3	3.1	4.0	4.6	6.3
Grade 4	<0.1	0.1	0.6	0.6
ATE/VTE	1.2	3.3	0.6	2.9
Grade 3	1.2	2.4	0.6	0.6
Grade 4	0	0.7	0	1.7
Grade 5	0	0.2	0	0.6
Proteinuria	1.0	1.5	2.3	4.0
Grade 3	1.0	1.4	2.3	2.9
Grade 4	0	0.1	0	1.1
Other hemorrhage	0.3	1.4	1.1	1.1
Grade 3	0.2	1.1	1.1	1.1
Grade 4	<0.1	0.2	0	0
Grade 5	0	0.1	0	0
Wound healing complications	0.3	0.6	0.6	1.1
Grade 3	0.2	0.3	0	0.6
Grade 4	0.1	0.2	0.6	0.6
Congestive heart failure	0.1	0.4	0	0.6
Grade 3	0.1	0.3	0	0
Grade 4	<0.1	<0.1	0	0
Grade 5	0	0.1	0	0.6
Gastrointestinal perforation	0	0.3	0	0
Grade 3	0	0.1	0	0
Grade 4	0	<0.1	0	0
Grade 5	0	0.1	0	0
Fistulae	0	0.1	0	0
Grade 3	0	0.1	0	0
CNS bleeding	0	<0.1	0	0
Grade 5	0	<0.1	0	0

*AVE/VTE* arterial or venous thromboembolism, *CNS* central nervous system.

#### Patients pretreated with taxanes for HER2-negative MBC

An exploratory analysis of data from the subpopulation of taxane-pretreated patients re-exposed to taxanes in the E2100 and AVADO trials (*n* = 311), with or without bevacizumab, demonstrated a benefit of adding bevacizumab (Miles et al. 2010b). Median PFS was 10.7 months with bevacizumab-taxane combination therapy versus 6.2 months with taxane alone (HR 0.533, *P* = 0.0001). The ORR was also significantly higher with combination therapy than with taxane monotherapy (49*%* vs. 27*%*; *P* < 0.005). OS was superior in patients receiving bevacizumab-taxane combination therapy; this was pronounced in the TNBC patient population with an OS of 25.6 months for taxane-bevacizumab therapy versus 15 months with taxane alone (HR 0.61; *P* = 0.0247). Despite promising results, this analysis has some limitations, notably a lack of stratification for taxane pretreatment in the E2100 trial and a limited number of taxane-pretreated patients in the AVADO trial receiving standard dose bevacizumab (*n* = 77). Furthermore, none of these trials was powered to show an OS benefit. However, the data suggest that patients previously treated with a taxane can benefit from retreatment with a taxane in combination with bevacizumab (Miles et al. 2010b). This might be valuable information, especially for patients with poor prognosis, such as those with TNBC.

The safety profile of bevacizumab in taxane-pretreated patients was consistent with the well-defined safety profile of bevacizumab in combination with taxane therapy.

#### HER2-positive BC

Recently, Phase II data were presented from an open-label study evaluating the efficacy and safety of bevacizumab in combination with trastuzumab and capecitabine as first-line therapy for HER2-positive LR/MBC. A total of 88 patients were enrolled and 40 were still on treatment at the time of reporting (Martin et al. 2012). Patients received bevacizumab (15 mg/kg on day 1) plus trastuzumab (8 mg/kg on day 1 of cycle 1, 6 mg/kg on day 1 of subsequent cycles) plus capecitabine (1,000 mg/m^2^ twice daily, days 1–14) every 3 weeks. The ORR (primary endpoint) was 73*%* (95*%* CI 62–82), comprising a CR and partial response rate of 7*%* and 66*%*, respectively. Median PFS was 14.4 months (95*%* CI 10.4 - not reached) with 33 events. Overall, 44*%* of patients experienced Grade ≥3 treatment-related AEs, and three patients discontinued capecitabine because of toxicity but continued with bevacizumab and trastuzumab. The combination of trastuzumab, bevacizumab, and capecitabine seems to be clinically active as first-line therapy for patients with HER2-positive LR/MBC (Martin et al. 2012). Ongoing Phase I and II trials are evaluating this combination.

#### Neoadjuvant treatment of HER2-negative and HER2-positive BC

GeparQuinto was a Phase III study evaluating bevacizumab in the neoadjuvant setting (von Minckwitz et al. 2012). In the HER2-negative component of this study, 1,948 patients were enrolled. A total of 144 patients (14.9*%*) who received epirubicin-cyclophosphamide followed by docetaxel and 176 patients (18.4*%*) treated with epirubicin-cyclophosphamide followed by docetaxel-bevacizumab had a pathological CR (pCR; pathological stage T0N0) (odds ratio with the addition of bevacizumab, 1.29, 95*%* CI 1.02 - 1.77, *P* = 0.04). The pCR achieved with the addition of bevacizumab increased to 20.5*%* if nodal involvement was included in the definition, to 21.7*%* if non-invasive residual disease in the breast was included, and to 24.6*%* if both were included. Among 663 patients with triple-negative tumors, the rates of pCR were 27.9*%* in the group that received epirubicin-cyclophosphamide followed by docetaxel and 39.3*%* in the group that received epirubicin-cyclophosphamide followed by docetaxel-bevacizumab (*P* = 0.003). Among 1,262 patients with hormone receptor-positive tumors, corresponding pCR rates were 7.8*%* and 7.7*%* (*P* = 1.00). The overall clinical response rate was higher in the bevacizumab group (87.4*%* vs. 79.6*%*), whilst the rate of breast-conserving surgery was identical in both groups (66.6*%*). A 4-week interval between the last bevacizumab dose and surgery was sufficient to reduce the incidence of therapy-associated surgical complications. Given the short follow-up period, these data cannot confirm that the observed increase in the rate of pCR can be translated into a survival advantage (von Minckwitz et al. 2012).

Similar results were seen in a study of patients with HER2-negative BC (*n* = 1,206), which investigated the effect of adding capecitabine or gemcitabine to docetaxel, compared with docetaxel alone, for four cycles, followed by treatment with doxorubicin-cyclophosphamide for four cycles with or without bevacizumab (15 mg/kg) for the first six cycles of chemotherapy (Bear et al. 2012). The addition of capecitabine or gemcitabine to docetaxel, compared with docetaxel alone, did not significantly increase the pCR rate (29.7*%* and 31.8*%*, respectively, vs. 32.7*%*; *P* = 0.69); while the addition of bevacizumab significantly increased pCR rate (28.2*%* without vs. 34.5*%* with bevacizumab; *P* = 0.02). The effect of bevacizumab was more pronounced in the hormone receptor-positive subset (15.1*%* without vs. 23.2*%* with bevacizumab; *P* = 0.007), with a weaker effect in the hormone receptor-negative subset (triple negative). The pCR rate was significantly increased when bevacizumab was added to docetaxel-capecitabine (36.1*%* vs. 23.5*%*; *P* = 0.009), but not when it was added to docetaxel-gemcitabine (35.8*%* vs. 27.6*%*; *P* = 0.10) or docetaxel alone (31.6*%* vs. 33.7*%*; *P* = 0.75). The addition of capecitabine or gemcitabine to docetaxel increased the overall AE rate as did the addition of bevacizumab to chemotherapy, particularly rates of hypertension, mucositis, and hand-foot syndrome (Bear et al. 2012).

Bevacizumab plus trastuzumab plus chemotherapy for primary inflammatory HER2-positive BC was evaluated in a Phase II, open-label, single-arm trial (BEVERLY-2) in 52 patients from 21 centers (Pierga et al. 2012). The combination achieved a pCR in 33 patients (63.5*%*, 95*%* CI 49.4 - 77.5). Neutropenia was the most common Grade 3 or 4 AE. Only one Grade ≥3 AE was regarded as related to bevacizumab (hypertension). Based on these results, the use of combination therapy with bevacizumab appears effective and well tolerated among patients with previously untreated inflammatory BC (Pierga et al. 2012).

The rate of surgical complications in the neoadjuvant setting was generally low, but was numerically higher in patients receiving bevacizumab than those who did not. Wound-healing complications occurred in ≤1.5*%* of patients in the bevacizumab arms versus ≤1*%* in the control arms. However, patients who undergo surgery ≤28 days prior to the last dose of bevacizumab have long been excluded from participating in clinical trials of bevacizumab (Hamilton & Blackwell 2011).

## Discussion

The addition of bevacizumab to chemotherapy has demonstrated ORR and PFS benefits in the first- and second-line treatment of MBC. However, according to a recent Cochrane Review, the overall benefit from adding bevacizumab to chemotherapy in MBC is considered modest. Benefits are dependent on the chemotherapy type and limited to a significant improvement in PFS and ORR with first- and second-line therapy (Wagner et al. 2012). Still, bevacizumab is an active and feasible treatment option for elderly patients and even for patients pretreated with taxanes. The benefit seems to be most pronounced for patients with TNBC, the subgroup that typically experiences the smallest benefit from standard chemotherapy. A trend towards an OS benefit with bevacizumab was seen in RIBBON-2 (pretreated patients) (Brufsky et al. 2012) and ATHENA (patients not previously treated for metastatic disease) (Pritchard et al. 2010). The effect on OS was persistent in the TNBC and elderly subgroups, but a clear OS benefit could not be shown (Thomssen et al. 2012;Biganzoli et al. 2012). However, ATHENA was a single-arm trial and it is difficult to draw conclusions regarding the impact of combining bevacizumab with chemotherapy on efficacy. Given the large sample size (*n* = 2,251), the trial provides valuable information on outcomes in clinically important subgroups, such as patients with TNBC and elderly patients. The lack of a clear OS benefit is in contrast to results from trials with bevacizumab in metastatic colorectal cancer where a significant difference in ORR, PFS, and OS was seen with the addition of bevacizumab to standard chemotherapy (Tappenden et al. 2007).

Preclinical studies have suggested accelerated tumor growth, local invasion, and distant metastasis after withdrawal of treatment with some antiangiogenic agents (Ebos et al. 2009; Pàez-Ribes et al. 2009). This could explain the lack of OS benefit associated with bevacizumab. However, a retrospective analysis of five Phase III, placebo-controlled, randomized trials investigating the efficacy and safety of bevacizumab in patients with metastatic breast, renal, colorectal, and pancreatic cancer does not support a decreased TTP, increased mortality, or altered disease progression patterns after cessation of bevacizumab therapy (Miles et al. 2011).

Bevacizumab has proven efficacy in the neoadjuvant setting; the increased rate of pCR was more pronounced in patients with hormone receptor-positive tumors. This result is encouraging, since patients with hormone-positive tumors tend to have low pCR with chemotherapy (von Minckwitz et al. 2012). Furthermore, it has been demonstrated that patients achieving a pCR achieve significantly longer disease-free survival and OS than non-responders (Feldman et al. 1986; Gralow et al. 2008; Verma et al. 2011).

Only occasionally severe AEs, such as bleeding or thrombosis (Phase II data), have been associated with bevacizumab. Overall, bevacizumab is well tolerated in combination with a range of chemotherapies and in a broad patient population, including elderly patients and those with a poor performance status. Data on bevacizumab-associated quality of life (QoL) are limited. Only the AVADO trial collected QoL data, but only to show that the addition of bevacizumab did not decrease patients’ QoL. Whether there is a bevacizumab-associated gain in QoL is unclear (Wagner et al. 2012).

Due to the lack of a clear survival benefit associated with bevacizumab and no QoL gain, the U.S. Food and Drug Administration recommended against bevacizumab in 2010 (National Cancer 2010). The question of what value PFS has in the context of MBC was addressed with the recommendation of including QoL measurements in future trials. Following this decision, a discussion regarding appropriate measures of clinical benefit began. In the era of multiple lines of subsequent therapy, PFS and TTP should be considered true endpoints because they are not affected by subsequent lines of chemotherapy. A review of 73 Phase III trials in MBC revealed that a strikingly small proportion of all trials (*n* = 9, 12*%*), and even fewer first-line trials (*n* = 4, 8*%*), demonstrated OS gains. A model proposed by Broglio and Berry (Broglio & Berry 2009), based on post-progression survival (PPS), suggests that with a short median PPS interval OS is an appropriate primary endpoint, but with a PPS >12 months it is not because of the greater likelihood that the effect on OS would be diluted by subsequent lines of therapy. This suggests that OS may be a more appropriate endpoint for second-line trials in which PPS is shorter, whereas PFS/TTP may be more appropriate in newly diagnosed disease with an expected longer PPS. Extrapolating this model to existing bevacizumab data we identified a trend towards an OS benefit in RIBBON-2, the only Phase III trial showing an OS benefit. Regarding a possible survival benefit, new data are awaited.

Given the numerous indications for bevacizumab, there is a need to investigate and validate putative biomarkers of efficacy. Concentrations of circulating VEGF before treatment were not associated with the efficacy of bevacizumab in an analysis of Phase III clinical trials (Jubb & Harris 2010). It was proposed that polymorphisms in components of the VEGF pathway could be used to predict benefit from bevacizumab (*VEGF*-2578AA and *VEGF*-1154AA) (Schneider et al. 2008). However, these data are not clear and have to be confirmed before entering clinical practice.

In conclusion, bevacizumab is an active treatment for patients with MBC. The addition of bevacizumab has shown a PFS and ORR benefit in the first- and second-line treatment of MBC, without a clear survival benefit. To date this effect has been most extensively studied and demonstrated in the subgroup of patients with triple-negative disease. Overall, bevacizumab is well tolerated with very few serious AEs, even for patients aged >70 years. There is a clear need for biomarkers/imaging techniques to assess treatment response and guide treatment, and future studies must be conducted.
